# Supplementary material for: Resolving the full spectrum of human genome variation using Linked-Reads
Source: Genome Res. 2019 Apr;29(4):635–45. doi: 10.1101/gr.234443.118 (PMC6442396; doi:10.1101/gr.234443.118)
Supplement: Supplemental Material [file supp_gr.234443.118_Supplemental_longranger-2.2.zip › longranger-2.2.tar.bz2!/lr2.2-public/lib/stan/cmdstan/stan/doc/api/html/lkj__cov_8hpp_source.html]

Stan: src/stan/prob/distributions/multivariate/continuous/lkj\_cov.hpp Source File


|  |  |
| --- | --- |
|  | Stan  2.5.0  probability, sampling & optimization |


- Main Page
- Related Pages
- Namespaces
- Classes
- Files

- File List
- File Members

All Classes Namespaces Files Functions Variables Typedefs Enumerations Enumerator Friends Macros Pages

- src
- stan
- prob
- distributions
- multivariate
- continuous

lkj\_cov.hpp

Go to the documentation of this file.

1 #ifndef STAN\_\_PROB\_\_DISTRIBUTIONS\_\_MULTIVARIATE\_\_CONTINUOUS\_\_LKJ\_COV\_HPP

2 #define STAN\_\_PROB\_\_DISTRIBUTIONS\_\_MULTIVARIATE\_\_CONTINUOUS\_\_LKJ\_COV\_HPP

3

4 #include <stan/prob/constants.hpp>

5 #include <stan/math/matrix.hpp>

6 #include <stan/math/matrix\_error\_handling.hpp>

7 #include <stan/math/error\_handling.hpp>

8 #include <stan/meta/traits.hpp>

9 #include <stan/prob/traits.hpp>

10

11 #include <stan/prob/distributions/univariate/continuous/lognormal.hpp>

12 #include <stan/prob/distributions/multivariate/continuous/lkj\_corr.hpp>

13

14 namespace stan {

15

16  namespace prob {

17

18  // LKJ\_cov(y|mu,sigma,eta) [ y covariance matrix (not correlation matrix)

19  // mu vector, sigma > 0 vector, eta > 0 ]

20  template <bool propto,

21  typename T\_y, typename T\_loc, typename T\_scale, typename T\_shape>

22  typename boost::math::tools::promote\_args<T\_y,T\_loc,T\_scale,T\_shape>::type

23  lkj\_cov\_log(const Eigen::Matrix<T\_y,Eigen::Dynamic,Eigen::Dynamic>& y,

24  const Eigen::Matrix<T\_loc,Eigen::Dynamic,1>& mu,

25  const Eigen::Matrix<T\_scale,Eigen::Dynamic,1>& sigma,

26  const T\_shape& eta) {

27  static const char\* function = "stan::prob::lkj\_cov\_log(%1%)";

28

29  using stan::math::check\_size\_match;

30  using stan::math::check\_finite;

31  using stan::math::check\_positive;

32  using boost::math::tools::promote\_args;

33

34  typename promote\_args<T\_y,T\_loc,T\_scale,T\_shape>::type lp(0.0);

35  check\_size\_match(function,

36  mu.rows(), "Rows of location parameter",

37  sigma.rows(), "columns of scale parameter",

38  &lp);

39  check\_size\_match(function,

40  y.rows(), "Rows of random variable",

41  y.cols(), "columns of random variable",

42  &lp);

43  check\_size\_match(function,

44  y.rows(), "Rows of random variable",

45  mu.rows(), "rows of location parameter",

46  &lp);

47  check\_positive(function, eta, "Shape parameter", &lp);

48  check\_finite(function, mu, "Location parameter", &lp);

49  check\_finite(function, sigma, "Scale parameter", &lp);

50  // FIXME: build vectorized versions

51  for (int m = 0; m < y.rows(); ++m)

52  for (int n = 0; n < y.cols(); ++n)

53  check\_finite(function, y(m,n), "Covariance matrix", &lp);

54

55  const unsigned int K = y.rows();

56  const Eigen::Array<T\_y,Eigen::Dynamic,1> sds

57  = y.diagonal().array().sqrt();

58  for (unsigned int k = 0; k < K; k++) {

59  lp += lognormal\_log<propto>(sds(k), mu(k), sigma(k));

60  }

61  if (stan::is\_constant<typename stan::scalar\_type<T\_shape> >::value

62  && eta == 1.0) {

63  // no need to rescale y into a correlation matrix

64  lp += lkj\_corr\_log<propto,T\_y,T\_shape>(y, eta);

65  return lp;

66  }

67  Eigen::DiagonalMatrix<T\_y,Eigen::Dynamic> D(K);

68  D.diagonal() = sds.inverse();

69  lp += lkj\_corr\_log<propto,T\_y,T\_shape>(D \* y \* D, eta);

70  return lp;

71  }

72

73  template <typename T\_y, typename T\_loc, typename T\_scale, typename T\_shape>

74  inline

75  typename boost::math::tools::promote\_args<T\_y,T\_loc,T\_scale,T\_shape>::type

76  lkj\_cov\_log(const Eigen::Matrix<T\_y,Eigen::Dynamic,Eigen::Dynamic>& y,

77  const Eigen::Matrix<T\_loc,Eigen::Dynamic,1>& mu,

78  const Eigen::Matrix<T\_scale,Eigen::Dynamic,1>& sigma,

79  const T\_shape& eta) {

80  return lkj\_cov\_log<false>(y,mu,sigma,eta);

81  }

82

83  // LKJ\_Cov(y|mu,sigma,eta) [ y covariance matrix (not correlation matrix)

84  // mu scalar, sigma > 0 scalar, eta > 0 ]

85  template <bool propto,

86  typename T\_y, typename T\_loc, typename T\_scale, typename T\_shape>

87  typename boost::math::tools::promote\_args<T\_y,T\_loc,T\_scale,T\_shape>::type

88  lkj\_cov\_log(const Eigen::Matrix<T\_y,Eigen::Dynamic,Eigen::Dynamic>& y,

89  const T\_loc& mu,

90  const T\_scale& sigma,

91  const T\_shape& eta) {

92  static const char\* function = "stan::prob::lkj\_cov\_log(%1%)";

93

94  using stan::math::check\_finite;

95  using stan::math::check\_positive;

96  using boost::math::tools::promote\_args;

97

98  typename promote\_args<T\_y,T\_loc,T\_scale,T\_shape>::type lp(0.0);

99  check\_positive(function, eta, "Shape parameter", &lp);

100  check\_finite(function, mu, "Location parameter", &lp);

101  check\_finite(function, sigma, "Scale parameter", &lp);

102

103  const unsigned int K = y.rows();

104  const Eigen::Array<T\_y,Eigen::Dynamic,1> sds

105  = y.diagonal().array().sqrt();

106  for (unsigned int k = 0; k < K; k++) {

107  lp += lognormal\_log<propto>(sds(k), mu, sigma);

108  }

109  if (stan::is\_constant<typename stan::scalar\_type<T\_shape> >::value

110  && eta == 1.0) {

111  // no need to rescale y into a correlation matrix

112  lp += lkj\_corr\_log<propto>(y,eta);

113  return lp;

114  }

115  Eigen::DiagonalMatrix<T\_y,Eigen::Dynamic> D(K);

116  D.diagonal() = sds.inverse();

117  lp += lkj\_corr\_log<propto,T\_y,T\_shape>(D \* y \* D, eta);

118  return lp;

119  }

120

121  template <typename T\_y, typename T\_loc, typename T\_scale, typename T\_shape>

122  inline

123  typename boost::math::tools::promote\_args<T\_y,T\_loc,T\_scale,T\_shape>::type

124  lkj\_cov\_log(const Eigen::Matrix<T\_y,Eigen::Dynamic,Eigen::Dynamic>& y,

125  const T\_loc& mu,

126  const T\_scale& sigma,

127  const T\_shape& eta) {

128  return lkj\_cov\_log<false>(y,mu,sigma,eta);

129  }

130

131

132  }

133 }

134 #endif

stan::is\_constant

Metaprogramming struct to detect whether a given type is constant in the mathematical sense (not the ...

**Definition:** traits.hpp:43

stan::scalar\_type

Metaprogram structure to determine the base scalar type of a template argument.

**Definition:** traits.hpp:138

traits.hpp

stan::prob::lkj\_cov\_log

boost::math::tools::promote\_args< T\_y, T\_loc, T\_scale, T\_shape >::type lkj\_cov\_log(const Eigen::Matrix< T\_y, Eigen::Dynamic, Eigen::Dynamic > &y, const Eigen::Matrix< T\_loc, Eigen::Dynamic, 1 > &mu, const Eigen::Matrix< T\_scale, Eigen::Dynamic, 1 > &sigma, const T\_shape &eta)

**Definition:** lkj\_cov.hpp:23

stan::math::check\_size\_match

bool check\_size\_match(const char \*function, T\_size1 i, const char \*name\_i, T\_size2 j, const char \*name\_j, T\_result \*result)

**Definition:** check\_size\_match.hpp:13

error\_handling.hpp

stan::math::check\_finite

bool check\_finite(const char \*function, const T\_y &y, const char \*name, T\_result \*result)

Checks if the variable y is finite.

**Definition:** check\_finite.hpp:52

matrix\_error\_handling.hpp

lkj\_corr.hpp

constants.hpp

traits.hpp

lognormal.hpp

stan::math::check\_positive

bool check\_positive(const char \*function, const T\_y &y, const char \*name, T\_result \*result)

**Definition:** check\_positive.hpp:54

matrix.hpp


---

[ Stan Home Page ]

*© 2011–2014,
Stan Development Team.*
